# Relationship Between Proactive Personality and Job Performance of Chinese Nurses: The Mediating Role of Competency and Work Engagement

**DOI:** 10.3389/fpsyg.2021.533293

**Published:** 2021-05-25

**Authors:** Xuehui Hu, Rong Zhao, Jing Gao, Jianzhen Li, Pei Yan, Xiaofei Yan, Shuai Shao, Jingkuan Su, Xiaokang Li

**Affiliations:** ^1^Xijing Hospital, Fourth Military Medical University, Xi’an, China; ^2^Scientific Research Department, Fourth Military Medical University, Xi’an, China

**Keywords:** proactive personality, job performance, competency, work engagement, structural equation model, nurse

## Abstract

**Background:** As one of the main participants in health care, nurses are esteemed an important driving force for the vigorous health care development. Studies report that nurses’ proactive personality has positive effects on their job performance; however, this relationship acquires further understanding.

**Objective:** A cross-sectional study was performed to explore the relationship between nurses’ proactive personality and job performance; the mediating role of nurses’ competency and work engagement in this relationship was also evaluated.

**Methods:** The study was performed in a large third-degree general hospital in October 2019, Xi’an, PR, China. A sample of 246 nurses participated in this cross-sectional study. Proactive personality was assessed with the Proactive Personality Questionnaire (PPS), job performance was assessed by Heilman three-item measurements, nurse competence was estimated with Nurse Competency Scale (NCS), and work engagement was assessed with the Utrecht Work Engagement Scale (UWES). The structural equation model was used to test the main hypotheses.

**Results:** Structural equation model analysis revealed that work engagement partially mediated the association between proactive personality and job performance. The serial two-mediator model which was used to explore the association between proactive personality and job performance through competency and work engagement, in sequence, was demonstrated.

**Conclusion:** This study demonstrates that work engagement partially mediated the association between nurses’ proactive personality and their job performance. The serial two-mediator model demonstrated that proactive personality was associated with job performance via competency and work engagement. This study also revealed the critical role of nursing managers in understanding the nurses’ proactive personality, which would facilitate them to enhance the latter’s competency and promote their work engagement. All these will in turn constantly improve the overall quality of nursing and advance professional development of nursing and benefits for patients.

## Introduction

Nurses are an important part of medical service. Nursing service and nursing performance improvement are based on nurses’ initiative, competence, and job engagement. Measures enhancing the nurses’ working efficiency can facilitate the hospital to gain a competitive edge in an increasingly intensive competition, which is also reflected as an aspect of comprehensive outcome of the entire staff job performance. Job performance is defined as the total work results generated by employees in a certain period based on their job responsibilities or task activities (Fried et al., 2008). Job performance is influenced by various factors, including organizational environment, job character, and job involvement, as well as certain individual characteristics. Proactive personality as an individual’s stable behavioral tendency can significantly affect his/her job performance (Kendig et al., 2017). The stressor–strain model (Park and Defrank, 2018) demonstrates the importance of proactive personality in work outcomes. As one of the multiple personality traits, proactive personality has attracted special attention of researchers and plays a critical role on people actively fulfilling task duties and positive consequences (Li et al., 2011; Bergeron et al., 2014; Park and Defrank, 2018). Proactive personality refers to the active behaviors that an employee took to overcome difficulties and obstacles, aiming to achieve organizational goals and complete tasks (Seibert et al., 1999). Chan (2006) found that highly proactive employees could, in the shortest time, get familiar with their work, establish a good working relationship at the workplace, integrate their efforts with others, and accomplish their work goals more easily. Crant J. (1995) showed that proactive personality showed a more powerful objectively predictive capacity on job performance than general intelligence parameters and positive consequences including individual performance (Bergeron et al., 2014; Park and Defrank, 2018).

There is a significant positive correlation between proactive personality and employee job performance (Kim et al., 2009; Fu and Rebecca, 2016). Proactive employees define their responsibilities more clearly, adapt themselves quickly to the work environment, and integrate themselves into the colleague team (Kammeyer-Mueller and Wanberg, 2003). Thompson (2005) believed that the relationship between proactive personality and job performance was regulated by employees’ network construction and their initiatives. Proactive employees affected both the objective and quantitative performance indicators as well as the subjective evaluation of supervisors. Fuller et al. (2018) showed certain intermediary behavioral variables, such as self-efficacy beliefs and anticipatory entrepreneurial cognition and playing mediated roles between proactive personality and individual job performance.

The competency theory (McClelland, 1973) claimed that competency was the core factor for improving job performance: the higher the competency, the better the job performance. Luna-Arocas and Morley (2015) defined competency as “a set of observable dimensions, including individual knowledge, skills, attitudes, and behaviors, as well as collective team, process, and organizational capabilities, that are linked to high performance and provide the organization with sustainable competitive advantage.” Hospital nurses’ competency for their job and their ability to maintain a higher performance level were closely related to their nursing quality and patient health outcomes. Na and Choi (2010) showed that nurse competence was positively correlated with job satisfaction and nursing performance; it also affected job satisfaction and nursing performance. Research indicated that proactive people could enhance their sense of competence and job performance (Gardner et al., 2011; Lin et al., 2014; Wei et al., 2018; Wu et al., 2018).

Work engagement stemmed from positive psychology which addresses the factors associated with normal and satisfactory activity, rather than those of mental disorders (Maslach and Leiter, 1997). Carlson et al. (2017) defined work engagement as one dynamic multidimensional variable, those observable behaviors associated with organizational goals and objectives. In addition, nurses’ work engagement enhanced their personal initiative, reduced the hospital mortality rate (Bargagliotti, 2012), and improved the hospital work quality significantly, and nurse managers could address relevant issues through positive psychology interventions (Bolier and Abello, 2014; Crane and Ward, 2016). Work engagement has been influenced by situational and disposition antecedents (Christain et al., 2011). Proactive personality was considered as a dispositional antecedent of work engagement and work engagement mediated the association of the effect of proactive personality on work engagement (Jawahar and Liu, 2017), job satisfaction (Li et al., 2017), and job performance (Bakker et al., 2012).

Basing on the theory of stressor-strain model and competency and above logical reasoning, our study investigated the potential mediator roles of competency and work engagement in the association between proactive personality and job performance among Chinese nurses. The association between proactive personality and job performance has been explored; however, few studies have simultaneously investigated the effects of competency and work engagement on this association. Thus, we hypothesized that competency and work engagement uniquely mediate the association between proactive personality and job performance. A serial model of two mediators (proactive personality → competency → work engagement → job performance) was verified.

## Materials and Methods

### Participants and Procedures

Nurses were enrolled through random selection from a third-grade general hospital in Shaanxi Province. According to the structural equation modeling criteria proposed by Jackson (2003), an appropriate sample size can avoid inaccurate estimation of standard errors and fitting indicators. It is suggested that an ideal sample size should meet the ratio of 20 cases per parameter to be estimated in the model or a less ideal ratio of 10 cases per parameter. Therefore, a total of 245 nurses participated in our study and the sample was composed by 245 eligible nurses, which included six males and 239 females. The participants’ average age was 31.50 ± 3.92 years. Thirty-three nurses had a working experience less than 5 years, 132 nurses had a working experience from 5 to 10 years, and 80 nurses had 10 or more years of working experience. Twenty-four participants had a junior college degree, 206 had a bachelor’s degree, and 15 had a master’s degree (10, 84, and 6%, respectively). All the participants were registered nurses, 202 were senior nurses, 41 were intermediate nurses, and 2 were senior nurses (82, 17, and 1%, respectively).

A total of 41 unit supervisors completed the survey. They come from the four departments of internal medicine, surgery, intensive care unit, and emergency and were selected to evaluate 245 nurses; all of them were female, with an average age of 43.93 ± 7.33 years. Five supervisors had a working experience less than 10 years, 25 supervisors had a working experience from 10 to 20 years, and 10 supervisors had a working experience from 20 to 30 years. Twenty-eight had a bachelor’s degree (68.29%), and 13 had a master’s degree (31.71%). All volunteer participants were fully informed of the study design, and their written consents were obtained. A questionnaire was distributed by the surveyors, and participants were instructed on the standard answering protocol. The participants’ average questionnaire completion time was around 15 min. The study was approved by the Ethics Committee of Xijing Hospital, the Fourth Military Medical University, Xi’an, China.

### Measures

#### Proactive Personality Questionnaire (PP)

The Chinese version of the Bateman and Crant (1993) Proactive Personality Scale (PPS) used in this study had 17 questions within a one-dimensional structure with Likert seven points scoring 1 – totally disagree, 2 – disagree, 3 – somewhat disagree, 4 – uncertain, 5 – somewhat agree, 6 – agree, and 7 – completely agree. The higher the score, the higher the level of individual initiative.

This Bateman PPS was used by Baba et al. (2011) to explore the relationship between proactive personality and job performance in 485 Chinese participants. The differences between Chinese and Western cultures did not show significant impact of the validation of this PPS reliability on the proactive personality structure. Most of the 10-item versions of the one-dimensional structure proactive personality questionnaire with good fitting in Chinese were adopted. In the current study, the Cronbach’s alpha coefficient is 0.90.

#### Job Performance (JP)

The unit supervisors were asked to provide a performance rating for each nurse. A Chinese version of the Heilman, Block, and Lucas (Heilman et al., 1992) measurement scale was used in this study. It includes three questions: “This employee is competent,” “This employee does his or her work effectively,” and “This employee does his or her work very well.” Each question was evaluated by Likert 5-level: “totally disagree,” “disagree somewhat,” “neutral,” “agree a little,” and “totally agree,” and assigned 1, 2, 3, 4, and 5 points, respectively. In our pilot study, the Chinese version was translated by a professional translator and validated by a small sample of participants. The results showed its capacity to evaluate employees’ performance. In the current study, the Cronbach’s alpha coefficient is 0.86.

#### Nurse Competency Scale (NCS)

Based upon the nursing competence framework proposed by Benner (1984), Meretoja et al. (2004) developed the Nurse Competence Power Table (NCS). The scale consists of 73 items in seven dimensions, which includes Helping role (1–8), Teaching coaching (9–23), Diagnostic functions (24–30), Managing situations (31–38), Therapeutic interventions (39–48), Performance of the work role (49–54), and Quality control capability (55–73). A Chinese version of NCS (Guo, 2015) was used in the current study.

The Visual Analogue Scale (VAS) was used to score each item, which ranged from 0 to 100, marked by a 10-cm straight line labeled “0” and “100” at both ends. Nurses rated their average nursing and clinical practice performance scale as 0∼25 for low competency, 26∼50 for competency, 51∼75 for good competency, and 76∼100 for a very good competency. The actual application frequency of each item adopts Likert 4-level ratings: “never used,” “rarely used,” “occasionally used,” and “often used,” and assigned 0, 1, 2, and 3 points, respectively. In the current study, the Cronbach’s alpha coefficient for the nurse competency scales is 0.96.

#### Utrecht Work Engagement Scale (UWES)

A shorter nine-item version of the Utrecht Work Engagement Scale (UWES-9, Schaufeli et al., 2006) was adopted to assess nurses’ work engagement. It includes three dimensions: “vigor,” “dedication,” and “absorption” and has a total of nine questions. Participants rated each item on a 7-point scale based on the frequency they adopt coping styles in their daily working (1 = never, 2 = several times a year or less, 3 = once a month or less, 4 = several times a month, 5 = once a week, 6 = several times a week 7 = every day). In the current study, the Cronbach’s alpha coefficient was 0.92. The internal consistency coefficients of the vigor, dedication, and absorption scales were 0.73, 0.82, and 0.85, respectively.

### Statistical Analysis

After data quality screening, the maximum likelihood estimation method was used to test the acceptability of a structural model for these data sets. Descriptive statistics and Pearson’s correlations were calculated for all measures. The mediating effect of competence and work engagement on the association between proactive personality and job performance was examined by structural equation modeling. Outlines and normality of all variables from the data set were analyzed before performing the structural equation model procedures. Skewness and kurtosis values were used to evaluate the normality of all the variables’ distribution (all ≤ ± 2). A two-step measurement model procedure was performed to examine the associations between latent variables and their respective indicators, as recommended by Anderson and Gerbing (1988). The proactive personality of the latent variables was formed using the method of item parcel. The measurement model consists of four potential factors and 16 observation variables. The model goodness of fit was assessed by Chi-square/df ratio (χ^2^/df), (Alan and Porter, 2001), the Comparative Fit Index (CFI; Bentler, 1990), the Goodness-of-Fit Indicator (GFI; Hu, 1995), and the Root Mean Square Error of Approximation (RMSEA; Yin et al., 2017). The Bootstrap estimation procedure (using the Monte Carlo method with 95% bias-corrected bootstrapped confidence interval method Mackinnon et al., 2004), was used to assess the association among all measures. All the analyses were performed by SPSS 22.0 and Mplus 7.4.

## Results

### Descriptive Statistics and Correlation

Descriptive statistics-calculated coefficients and correlations for all the measured variables are presented in Table 1. Proactive personality (PP) was significantly and positively associated with competency, work engagement, and job performance; competency was also significantly and positively associated with work engagement and job performance; and work engagement is significantly and positively related to job performance.

**TABLE 1:** Descriptive statistics and zero-order correlations for all measures (*N* = 245).

	**Mean**	**SD**	**PP**	**CO**	**WE**	**JP**
1. PP	5.461	0.873	–			
2. CO	2.522	0.380	0.342^**^	–		
3. WE	5.76	1.105	0.388^**^	0.302^**^	–	
4. JP	4.48	0.586	0.434^**^	0.280^**^	0.545^**^	–

^**^p < 0.01. PP, proactive personality; CO, competency; WE, work engagement; JP, job performance.

### Measurement Model

The measurement model consisted of four latent variables and 16 measurement indices. Confirmatory factor analysis of the model showed that the model was well supported by data set: χ^2^ (98, *N* = 245) = 195.411, *p* < 0.01; RMSEA = 0.064, 90% confidence interval (CI) = 0.051–0.077; CFI = 0.964; TLI = 0.956 and SRMR = 0.040. All the factor loadings for the indicators of the latent variables were significant (*p* < 0.01), indicating that all the latent factors were well represented by their respective indicators. All the intercorrelations among the latent variables were significantly correlated in a conceptually expected manner (*p* < 0.01).

### Structural Model

**FIGURE 1:**
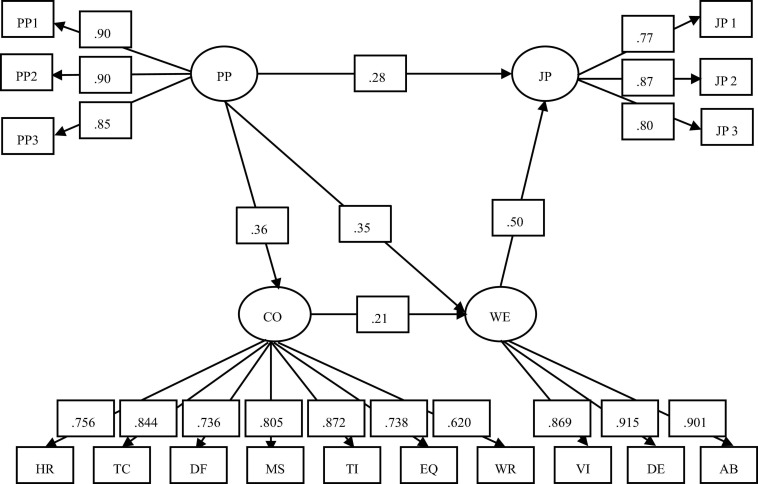
The mediation model for the relationship between Proactive Personality and Job Performance of Chinese nurses (*N* = 245). Factor loading is standardized and significant at the 0.01 level. PP1–PP3, three parcels of Proactive Personality (PP); HR, helping role; TC, teaching-coaching; DF, diagnostic functions; MS, managing situations; TI, therapeutic interventions; EQ, ensuring quality; WR, work role; HR, TC, DF, MS, TI, EQ, and WR are subscales of competency (CO); VI, vigor; DE, dedication; AB, absorption; VI, DE, and AB are subscales of work engagement (WE); and JP1–JP3, three parcels of Job Performance (JP).

To test our hypotheses, we built a mediated model with two mediators (competency and work engagement) to explore both direct and indirect pathways from proactive personality to job performance. The results showed that the structural model was supported by model fitness: χ^2^ (98, *N* = 245) = 195.411, *p* < 0.01; RMSEA = 0.064, (90% confidence interval [CI] = 0.051 to 0.077); CFI = 0.964; TLI = 0.956 and SRMR = 0.040. However, as the coefficient of path from competency to job performance (β = 0.056, *p* = 0.40) was not significant, we deleted this path and conducted model fitting analysis again. The structural model demonstrated its acceptability based on data set: χ^2^ (99, *N* = 245) = 196.205, *p* < 0.01; RMSEA = 0.063 (90% confidence interval [CI] = 0.050 to 0.076); CFI = 0.964; TLI = 0.957 and SRMR = 0.042. The final model is shown in Figure 1. Figure 1 reveals the direct and mediating paths in the model. Proactive personality shows the partially mediational effect on job performance via work engagement. In addition, the path from proactive personality to job performance was also partially mediated by the serial mediators of competency and work engagement.

### Assessment of Mediation

Bootstrapping procedures were conducted to examine the significance of the indirect effects. Five thousand bootstrap samples were generated using random sampling. The results are shown in Table 2. The indirect effect of proactive personality and job performance through work engagement was significant, as well as the chain mediating effect of competency and work engagement. The results showed that the path coefficients from proactive personality to competency (β = 0.282, *p* < 0.01) and work engagement (β = 0.368, *p* < 0.01) were significantly positive. The path coefficients from proactive personality to job performance (β = 0.352, *p* < 0.01), the path coefficients of mediator competency to work engagement (β = 0.215, *p* < 0.01), and the path coefficients of work engagement to job performance (β = 0.505, *p* < 0.01) were also significant. The proportion of both indirect effects (PP →WE→JP and PP →CO→WE→JP) to the total effects is 44%.

**TABLE 2:** Standardized indirect effects and 95% confidence intervals for the model (*N* = 245).

**Model pathways**	**Estimated effects**	**95% CI**	
		**Lower**	**Upper**
PP →WE→JP	0.18^a^	0.09	0.26
PP →CO→WE→JP	0.04^a^	0.01	0.07

*^*a*^Empirical 95% confidence interval does not overlap with zero.*

## Discussion

The study explores the effects of competency and work engagement on the association between proactive personality and job performance among Chinese nurses. Our results demonstrate that proactive personality has both direct and indirect effects on job performance. Work engagement partially mediated the link between proactive personality and job performance. In addition, our results unravel that proactive personality could influence job performance via the chain-mediating effects of competency–work engagement.

Our study showed that work engagement played a significant mediating role between proactive personality and job performance. Individuals with proactive personality are more inclined to actively seek opportunities and take positive actions to seek meaningful changes (Buss, 1987; Crant J., 1995; Crant J.M., 1995). Our study is a further extension of previous research findings, providing strong evidence and possible reasons why proactive personality has a positive promoting effect on job performance. Competency indirectly affects job performance by work engagement, because workers improve their own work ability proactively which can promote the work needs and resources to adapt to their own ability and needs (Bakker et al., 2012). Cultivating work engagement of individuals with a proactive personality will contribute to their job performance. The high coefficients of individuals’ work engagement and job performance implied that enhancing work engagement could contribute to a high job performance improvement; regarding proactive personality, individuals tend to be ready to contribute and put more energy in and be more dedicated to their work so as to achieve higher job performance as far as possible (Owens et al., 2016).

Our study also demonstrated that proactive personality could account for job performance through competency and work engagement. This is similar to Ford’s (2011) servery, who argued that better competency is more likely to develop when proactive personality is present, and competency has a positive effect on work engagement and job performance. Nurses with high competency are more capable of experiencing the value and pleasure of work, which may trigger a more active engagement in work. In contrast, a nurse with a low competency and little professional knowledge and skills was likely to generate negative emotions and reduce their work engagement in work (Michihiro et al., 2006). Moreover, some studies showed that competency could promote individual job satisfaction, but the mechanism of how competency affects job satisfaction remains to be elucidated. Therefore, to understand the intermediary mechanism of competency affecting job satisfaction was of great necessity and significance (Numminen et al., 2016). By verifying the mediating effect of competency and work engagement on job performance, our study implied that nursing managers could prioritize the core issue of job performance from various perspectives and improve nurse job performance through diversified ways.

Basing on our results, we proposed some suggestions on enhancement for nurses’ job performance.

First, our study stresses the importance of proactive personality because it can positively affect the nurses’ competency, work engagement, and job performance. On the one hand, during nurse recruitment, in addition to rigid indexes such as education background and level and professional skills, their personality traits are evaluated, especially giving priority to those with proactive personality; on the other hand, the nurses’ proactive personality are actively cultivated by organizing lively and interesting group activities to attract nurses to be actively engaged and to improve their sense of initiative, provide nurses with opportunities to participate in various activities, turn their reasonable suggestions into reality, help them experience the fun of initiating, and increase their initiative experience.

Nurses should be encouraged to take the initiative to learn more professional knowledge and skills, actively ask for help when encountering difficulties, and be given positive feedback to improve their proactive behaviors. Basing on the Person-Job fit theory and the importance of proactive personality in a working environment (Chan, 2006), managers should recognize one’s outstanding ability and personality traits to arrange his/her job or assign him/her tasks appropriate to him/her. Besides, the nursing manager should take measures to create a friendly environment for nurses to release their proactive personality potentials, for example, providing organizational support (Li et al., 2014), adopting an appropriate leadership style (Yin et al., 2017), and building a fair organizational environment.

The study demonstrated the mediating effect of competency and work engagement on job performance; it suggested that administrators should give great consideration to nurses’ competence and improve the job work engagement of nurses in order to improve their job performance. Some studies found that the common factors affecting work engagement were age, education, and marital status (Simpson, 2009); organizational identity and external work environment (Spence Laschinger et al., 2006); leadership style (Manning, 2016); and sense of organizational support (Ravangard et al., 2015). Therefore, in order to enhance the work engagement of nurses, we can take measures according to these factors. For example, nursing managers can learn how to appropriately authoritatively support and cultivate the followers and provide them with the opportunity to participate in decision-making. Another suggestion to nursing managers is to actively improve nurse competence; administrators should pay more attention to the continuing-education training demands of nurses at different stages of their careers. The combination of modern information technology and other means to develop learning resources for nurses should be made, so that they can learn more professional knowledge and skills, broaden their horizons, and constantly tap their potentials. Leaders can also provide nurses with appropriate posts, which may enhance nurses’ perception of person–job fit and cultivate their competency. At the same time, Cooper-Thomas et al. (2014) found that nurses with a higher level of competency can further strengthen individual work engagement, promote each other, and facilitate the development of job performance.

There are some limitations in this study. First, the research design was a cross-sectional study with a small sample size and only in one general third-grade hospital; the effect of demographic variables was hard to balance and control. Therefore, the longitudinal and experimental design with a larger sample size is our next study. It can provide a more comprehensive validation, and understanding the work performance and relevant factors and categorizing nurses from different career levels will help in the formulation of targeted intervention measures. Second, the data was collected through self-report measures which were a threat to internal validity and cause self-interest bias. Future studies could integrate multiple assessment methods and use longitudinal approaches or experiments to examine the causal associations in our model. Third, variables which predict work engagement and job performance were not addressed in this study, which need further studies; for example, China’s national conditions and culture may influence specific variables such as leadership style and organizational support, which can be incorporated into the research model to enrich the theoretical aspect of the influencing mechanism of job performance.

## Conclusion

The current study sheds light on the mechanism between proactive personality, competency, work engagement, and job performance among Chinese nurses. These results highlight the importance of competency and work engagement to the influence of proactive personality and job performance. It could provide nursing managers a new viewpoint on how to improve nurse job performance from several aspects, including paying attention to the proactive personality traits, competency, and work engagement, which will increase nurses’ work enthusiasm and initiative, constantly improve the overall quality of nurses service, and promote the development of the nursing career.

## Data Availability Statement

The datasets generated for this study are available on request to the corresponding authors.

## Ethics Statement

The studies involving human participants were reviewed and approved by the Ethics Committee of the Fourth Military Medical University. The patients/participants provided their written informed consent to participate in this study.

## Author Contributions

XH and RZ designed the research. JS and XL guided and reviewed the research. JG and PY carried out the investment. XY analyzed the results. XH, JG, JL, and SS wrote the manuscript. All authors contributed to the article and approved the submitted version.

## Conflict of Interest

The authors declare that the research was conducted in the absence of any commercial or financial relationships that could be construed as a potential conflict of interest.
